# Aerobic exercise combined with FK866 ameliorates Alzheimer’s disease-like pathology in APP/PS1 mice with NAMPT abnormality

**DOI:** 10.3389/fimmu.2026.1876444

**Published:** 2026-07-24

**Authors:** Chang Sun, Tinggui Zhang, Bei Fan, Xianliang Zhang, Na Zhao

**Affiliations:** 1 College of Sports and Health, Shandong Sport University, Jinan, China; 2 Department of Rehabilitation Medicine, Jinan Hospital, Jinan, China; 3 School of Physical Education, Shandong University, Jinan, China

**Keywords:** aerobic exercise, Alzheimer’s disease, mitochondrial dysfunction, NAD+, NAMPT, neuroinflammation

## Abstract

Nicotinamide phosphoribosyltransferase (NAMPT), the rate-limiting enzyme in the NAD^+^ salvage pathway, has emerged as an important regulatory node linking metabolic homeostasis to neuroinflammatory responses. However, although pharmacological inhibition of NAMPT may attenuate inflammatory activation, it may also reduce intracellular NAD^+^ availability, thereby exacerbating mitochondrial dysfunction and disturbances in energy metabolism. Here, we found that 6-month-old APP/PS1 mice exhibited an abnormal, cell type-specific distribution of NAMPT in the brain, characterized by reduced neuronal NAMPT and increased microglial NAMPT. This abnormality was accompanied by disrupted NAD^+^ homeostasis, marked neuroinflammatory activation, mitochondrial structural and functional impairment, increased β-amyloid (Aβ) burden, and impaired learning and memory ability. FK866 (1 mg/kg per injection, once every other day for 12 weeks) alone exerted limited effects and was insufficient to ameliorate the overall pathological phenotype. By comparison, a 12-week aerobic treadmill exercise program markedly ameliorated cognitive impairment, attenuated Aβ pathology, suppressed neuroinflammation, and improved mitochondrial integrity and bioenergetic function in APP/PS1 mice. More importantly, under NAMPT-inhibited conditions, exercise still preserved hippocampal NAD^+^ homeostasis and maintained significant neuroprotective effects. Compared with exercise alone, the combined intervention with exercise and FK866 further improved selected indices related to Aβ pathology, neuroinflammation, oxidative stress, and mitochondrial damage. These additional effects may be associated with increased NAD^+^ biosynthesis through the NMNAT3 pathway, as well as reduced NAD^+^ consumption and neuroinflammatory activation through the suppression of CD38 and PARP1 expression.

## 1 Introduction

Nicotinamide phosphoribosyltransferase (NAMPT) is the rate-limiting enzyme in the NAD^+^ salvage pathway and plays an important role in maintaining NAD^+^ homeostasis. Recently, growing evidence has linked NAD^+^ to neuroinflammatory processes in neurodegenerative diseases (1). Studies have shown that changes in NAD^+^ levels not only affect cellular energy metabolism but also participate in regulating inflammatory responses. In particular, in inflammation-related cells such as microglia, NAD^+^ metabolic remodeling has been closely associated with inflammatory activation (2). Previous studies have shown that depletion of NAD^+^ in microglia can significantly ameliorate the progression of inflammation-mediated diseases (3). In addition, NAMPT has been implicated in microglial activation and neuroinflammatory processes in mouse models of ischemic neural injury, whereas inhibition of NAMPT has been shown to alleviate neuroinflammation (4). These findings suggest that NAMPT is not merely a metabolic enzyme required for maintaining NAD^+^ supply but may also represent an important regulatory node linking metabolic homeostasis to neuroinflammatory responses.

Based on the role of NAMPT in inflammatory regulation, FK866, a specific inhibitor of NAMPT, has recently received considerable attention in studies related to neural injury. Previous studies have shown that FK866 can attenuate glial activation and reduce the release of inflammatory cytokines in models of traumatic brain injury and cryoinjury, suggesting its potential anti-inflammatory effects (4, 5). However, as the key rate-limiting enzyme for NAD^+^ biosynthesis, NAMPT is widely involved in multiple cellular processes, including DNA repair, neuroinflammation, immune regulation, and mitochondrial energy metabolism. Studies have also shown that, while inhibiting NAMPT, FK866 may reduce intracellular NAD^+^ levels, further induce mitochondrial dysfunction and disturbances in energy metabolism, and even contribute to neurodegenerative changes (6). Therefore, the effects of FK866 are not limited to the suppression of inflammation-related metabolic processes but may also interfere with physiological NAD^+^ requirements, thereby limiting its clinical application.

Alzheimer’s disease (AD) is a common neurodegenerative disease characterized by increased Aβ plaque deposition and progressive decline in learning and memory ability (7). Recent studies have shown that AD pathology is also accompanied by persistent neuroinflammatory activation and impaired energy metabolism (8). Accumulating evidence has demonstrated that neuroinflammation plays an important role in the pathogenesis of AD. Cluster of differentiation 38 (CD38) and poly(ADP-ribose) polymerase 1 (PARP1) are important NAD^+^-consuming enzymes involved in the regulation of NAD^+^ homeostasis. Studies have also shown that suppressing CD38- and PARP1-associated NAD^+^ depletion can ameliorate neuroinflammatory responses in the AD brain (9–11), suggesting that targeting NAD^+^ metabolism may represent a promising therapeutic strategy for improving neuroinflammation in AD. Notably, decreased NAD^+^ levels in the AD brain are closely associated with mitochondrial dysfunction and neuronal damage. Previous studies have shown that a decline in brain NAD^+^ levels may reduce the activity of mitochondrial respiratory chain complexes, decrease ATP production, aggravate oxidative stress, and disrupt mitochondrial membrane integrity, thereby leading to mitochondrial damage (12). Previous work has shown that long-term FK866 treatment reduced neuronal NAD^+^ levels and aggravated AD pathology. These findings suggest that maintaining NAD^+^ at an appropriate level is critical for the treatment of AD. Therefore, reducing the adverse effects associated with NAMPT inhibition may be of considerable importance for the clinical translation of NAMPT-targeted strategies in AD.

Recent studies have shown that, unlike single drugs that mainly act on a specific molecular target, long-term regular exercise, as a systemic physiological intervention, not only suppresses the expression of proinflammatory cytokines such as IL-1β, IL-6, and TNF-α (13), but also exerts broader regulatory effects. More importantly, exercise regulates NAD^+^-dependent mitochondrial protective pathways and maintains metabolic homeostasis by modulating NAD^+^-related metabolic enzymes (14, 15). These findings suggest that exercise may provide a relatively stable NAD^+^ metabolic environment during FK866 treatment, thereby attenuating its detrimental effects on neuronal energy metabolism while preserving its anti-inflammatory effects.

Based on this, in the present study, we aimed to use APP/PS1 mice and their wild-type littermates to investigate the effects of exercise combined with FK866 on cognitive function, hippocampal Aβ pathology, neuroinflammation, mitochondrial function, and NAD^+^ metabolism in APP/PS1 mice.

## 2 Materials and methods

### 2.1 Animals

From Aniphe Biolaboratory Inc. (Nanjing, China), we acquired male APPswe/PS1dE9 (APP/PS1) double transgenic mice and non-transgenic C57BL/6 wild-type (WT) mice. All animals were housed under conventional conditions (22 ± 2 °C, 40–60% relative humidity). Mice were housed singly in plastic cages and allowed to feed and drink ad libitum. All animals were maintained on a 12 h light/12 h dark cycle with the light onset at 06:00. All animal protocols were approved by the institutional Animal Welfare and Use Committee.

At 3 months of age, male APP/PS1 transgenic mice and male WT littermate controls were randomly assigned to different treatment groups: Wild type control group (WT, n=15), APP/PS1 transgenic saline control group (AD-saline, n=15), APP/PS1 transgenic saline exercise intervention group (AD-EXE-saline, n=15),APP/PS1 transgenic FK866 intervention group (AD-FK866, n=15), and APP/PS1 transgenic FK866 and exercise combined intervention group (AD-EXE-FK866, n=15). Animal protocols were conducted in accordance with the Animal Welfare and Use Committee of East China Normal University (approval code m20210407).

### 2.2 Exercise and FK866 intervention protocol

Mice in the exercise groups began treadmill training at 3 months of age, following a two-phase protocol consisting of an adaptation period and a formal training phase. According to Baker (16), treadmill running at 12 m/min in mice corresponds to approximately 45%–55% of maximal oxygen uptake, representing a moderate-intensity aerobic exercise. In addition, previous studies have demonstrated that long-term treadmill exercise at this intensity, including a 12-week intervention protocol, can effectively prevent or ameliorate AD-like pathological changes in APP/PS1 mice (17, 18). Therefore, this established moderate-intensity aerobic exercise protocol was used in the present study. The adaptation period lasted for 1 week and occurred daily from 17:00 to 18:00. During this period, mice were exercised at speeds of 5 m/min, 8 m/min, and 12 m/min, for two consecutive days at each speed. The seventh day was designated as a rest day.

Formal training lasted for 12 weeks. Each training session lasted for 45 min and was conducted 5 days a week. The warm up period of each training day was 5 min at 5 m/min, 5 min at 8 m/min, 30 min at 12 m/min, and the cool down period was 5 min at 5 m/min. For the purpose of eliminating possible environmental effects, the non-exercise group mice were also exposed to a stationary treadmill for 12 weeks, with the same duration as the exercising group.

FK866 (MedChemExpress, HY-50876) was administered intraperitoneally starting at 3 months of age at 1 mg/kg per injection, once every other day for 12 weeks (19). This dosing regimen was selected with reference to previous studies and our previous experimental protocol in which FK866 was used to inhibit NAMPT *in vivo* (20). Mice in the control groups received equal-volume intraperitoneal injections of 0.9% normal saline on the same schedule.

### 2.3 Morris water maze test

Morris water maze (MWM) test has 5 days spatial acquisition and 1 day spatial probe trial. In the spatial acquisition, we observed the spatial learning ability of mice in each group by the escape latency, the percentage of time spent in the platform quadrant and the percentage of swimming distance in platform quadrant for 5 days. On the sixth day, the platform was removed and the spatial probe trial was performed. For each mouse, it was allowed to enter the tank from each quadrant one by one from the opposite quadrant, each time for 60 seconds. The spatial memory was detected by video tracking system by the frequency of platform crossing and the proportion of time spent in platform quadrant.

### 2.4 Tissue preparation

For tissue collection (n = 15 per group), mice were fasted for 12 h and euthanized 24 h after the completion of the Morris water maze test. Briefly, mice were placed in an induction chamber and euthanized by an overdose of inhaled isoflurane (≥5% isoflurane in oxygen) until respiratory arrest was observed. Cervical dislocation was then performed as a secondary method to ensure death. After death was confirmed, brains were rapidly removed, and the hippocampi were dissected on ice. Hippocampal tissues from six mice were used for ELISA assays and Western blotting, and the corresponding contralateral hippocampus was used for immunohistochemical assay. Hippocampus from another six mice was used for RNA isolation and further qPCR. Hippocampi from the rest three mice were used for TEM and immunofluorescence staining.

### 2.5 Immunohistochemistry

Immunohistochemical staining was performed following previous reports (21). Briefly, dissected one cerebral hemisphere of each mouse was immersed in 4% buffered formaldehyde for overnight fixation at 4 °C and then embedded in paraffin. Chosen were three slices from the interval 1.70–2.70 mm caudal to the bregma and coronally sectioned at 6 μm thickness. These sections were blocked with normal mouse serum and then incubated with a primary antibody against Aβ (Abcam, ab2539; 1:1000) overnight at 4 °C. Thereafter, the sections were incubated with secondary antibodies of goat anti-rabbit IgG (Abcam, ab150077; 1:500).

Images were acquired under a NIKON DS-U3 microscope. Images were acquired using a Nikon microscope equipped with a DS-U3 imaging system under identical imaging conditions. For quantification, hippocampal sections were photographed under identical imaging conditions and analyzed using Image-Pro Plus 6.0 software. After background correction, Aβ-positive staining was identified using the same threshold for all images, and the integrated optical density (IOD) and positive staining area were measured. Aβ immunoreactivity was expressed as average optical density (AOD), calculated as IOD divided by the positive staining area. The mean AOD value from three sections of each mouse was used for statistical analysis.

### 2.6 Immunofluorescence staining

Immunofluorescence staining was performed to assess the activation level of microglia by measuring ionized calcium-binding adapter molecule-1 (Iba-1) and nicotinamide phosphoribosyltransferase (NAMPT) abundance within the hippocampus. Briefly, paraffin-embedded brain tissues were sectioned into 4 μm slices, with one slice collected every 60 μm. For each mouse, three slices were selected per indicator. Paraffin sections underwent deparaffinization and rehydration, followed by antigen retrieval, circle marking, autofluorescence quenching, and blocking. Tissue sections underwent sequential incubation, first with primary antibodies overnight at 4 °C, then with the appropriate secondary antibodies at ambient temperature. After nuclear counterstaining with 4′, 6-diamidino-2-phenylindole (DAPI), sections were mounted and subsequently imaged under a microscope. The primary antibody used was anti-Iba-1 (Affinity, DF6442; dilution 1:50) and anti-NAMPT (Affinity, DF6059; dilution 1:50). For each slice, three visual fields within the hippocampal area were randomly selected, and quantitative analysis of Iba-1 and NAMPT-positive staining was performed using Image-Pro Plus software (version 6.0).

### 2.7 Transmission electron microscopy

TEM was performed using protocols adapted from prior publications (22). After anesthesia and perfusion as previously detailed, one cerebral hemisphere from each brain was promptly immersed in ice-cold 3% glutaraldehyde (pH 7.4). The tissue was subsequently post-fixed in 1% osmium tetroxide for two hours at ambient temperature, embedded in EPON 812 resin, and trimmed into ~1 mm³ blocks for transmission electron microscopy (Hitachi HT7700). Ultrathin sections (~70 nm) prepared from the hippocampal CA1 area were selected for downstream analyses. Anatomically, CA1 comprises roughly one-third of the dorsal and middle portions of the hippocampus and is bounded from the DG by the hippocampal fissure. For each animal, three tissue sections were examined, and within every section three microscopic fields were selected to tally the number of damaged mitochondria per field. Damage was operationally defined as disrupted cristae, organellar swelling, or intramedullary lesions characterized by abundant vacuolation.

### 2.8 Real-time PCR

Relative mitochondrial-to-nuclear DNA content was calculated from hippocampal samples by quantitative real-time PCR. Total DNA from the hippocampus was extracted using TRIzol reagent (B610409-0025; Sangon Biotech, Shanghai, China). The primers for the oligonucleotide pairs were provided by Sangon Biotech (Shanghai, China), and are summarized in **Table 1**. Quantitative PCR was then performed using Hieff^®^ qPCR SYBR Green Master Mix (11203ES08; Yeasen Biotechnology, Shanghai, China) with β-actin as a reference gene. The relative transcript level was calculated using the 2−ΔΔCt method.

**Table 1:** Primer pairs used for qPCR experiment.

Gene	Forward primer	Reverse primer
*mtDNA*	CAAACCTACGCCAAAATCCA	GAAATGAATGAGCCTACAGA
*nDNA*	ACGGACCAGAGCGAAAGCA	GACATCTAAGGGCATCACAGAC
*β-actin*	CCTCTATGCCAACACAGTGC	CCTGCTTGCTGATCCACATC

### 2.9 Enzyme-linked immunosorbent assay

Levels of Aβ_42_(Elabscience,E-EL-M30010),Aβ_40_(Elabscience,E-EL-M3009),IL-10 (Abcam,ab310329),TNF-α(Abcam,ab183218),IL-6(Abcam,ab314470),IL-1β(Abcam,ab283818), Complex I(mlbio,ml058155), Complex IV(mlbio,ml058158), ATP(mlbio,ml076315),H_2_O_2_(mlbio,ml009876-1),NAD^+^/NADH(Beyotime, S0175, Shanghai, China)in hippocampal tissue were measured using commercial ELISA kits according to the manufacturers’ instructions. Briefly, equal amounts of protein were added to each well. Absorbance was measured at 450 nm by a microplate reader. Concentrations were calculated by interpolation from a calibration curve.

### 2.10 Western blotting

Hippocampal tissues were rinsed with ice-cold PBS and homogenized on ice in pre-chilled RIPA lysis buffer (Beyotime Biotechnology, P0013B) supplemented with a protease/phosphatase inhibitor cocktail. Briefly, ~20 mg hippocampal tissue was homogenized in 200 μL lysis buffer using a bead-based tissue homogenizer (three metal beads per tube), followed by incubation on ice for 20 min. Lysates were centrifuged at 12,000 × g for 10 min at 4 °C, and supernatants were collected. Protein concentrations were determined using a BCA protein assay kit (Beyotime Biotechnology, P1081).

Equal amounts of protein were mixed with SDS sample loading buffer and denatured at 95 °C for 5 min, separated by SDS–PAGE, and transferred onto PVDF membranes. Membranes were blocked with 5% non-fat milk in TBST (Tris-buffered saline containing 0.1% Tween-20) for 1 h at room temperature and incubated overnight at 4 °C with the following primary antibodies: NMNAT1 (Cell Signaling Technology, #98354, 1:1000), NMNAT2 (Affinity, DF13581, 1:1000), NMNAT3 (Abcam, ab121030, 1:2000), PARP1 (Affinity, DF7198, 1:1000), and CD38 (Abcam, ab216343, 1:2000). After washing with TBST, membranes were incubated with HRP-conjugated secondary antibodies for 1 h at room temperature: goat anti-rabbit IgG (H+L) HRP (Affinity, S0001, 1:3000) or goat anti-mouse IgG (H+L) HRP (Affinity, S0002, 1:3000). Immunoreactive bands were visualized using enhanced chemiluminescence (ECL) reagents and captured with a digital imaging system.

Band intensities were quantified using ImageJ. Target protein expression levels were normalized to the corresponding loading control (β-tubulin: Affinity, DF7967, 1:1000) and expressed as relative fold changes compared with the designated control group.

### 2.11 Statistical analysis

All statistical analyses were performed using GraphPad Prism software (version 10.0, La Jolla, CA, USA) and IBM SPSS Statistics software (version 15.0, Chicago, IL, USA). All values are presented as means ± standard error (Mean ± SEM). The significance level was P< 0.05. WT-saline vs AD-saline were compared by an independent samples t-test. Group differences were analyzed by two-way analysis of variance (ANOVA) with exercise and FK866 treatment as the main effects. *Post hoc* t-tests were performed to analyze significant ANOVA results. Exclusion criteria were predefined before data analysis; animals or samples were excluded only in cases of death during the intervention, failure of tissue collection or assay due to technical reasons, or poor sample quality precluding reliable measurement, and no data points were excluded on the basis of the results.

## 3 Results

### 3.1 Abnormal cell type-specific distribution of NAMPT in the hippocampus of APP/PS1 mice is accompanied by NAD^+^ metabolic imbalance

**Figure 1:**
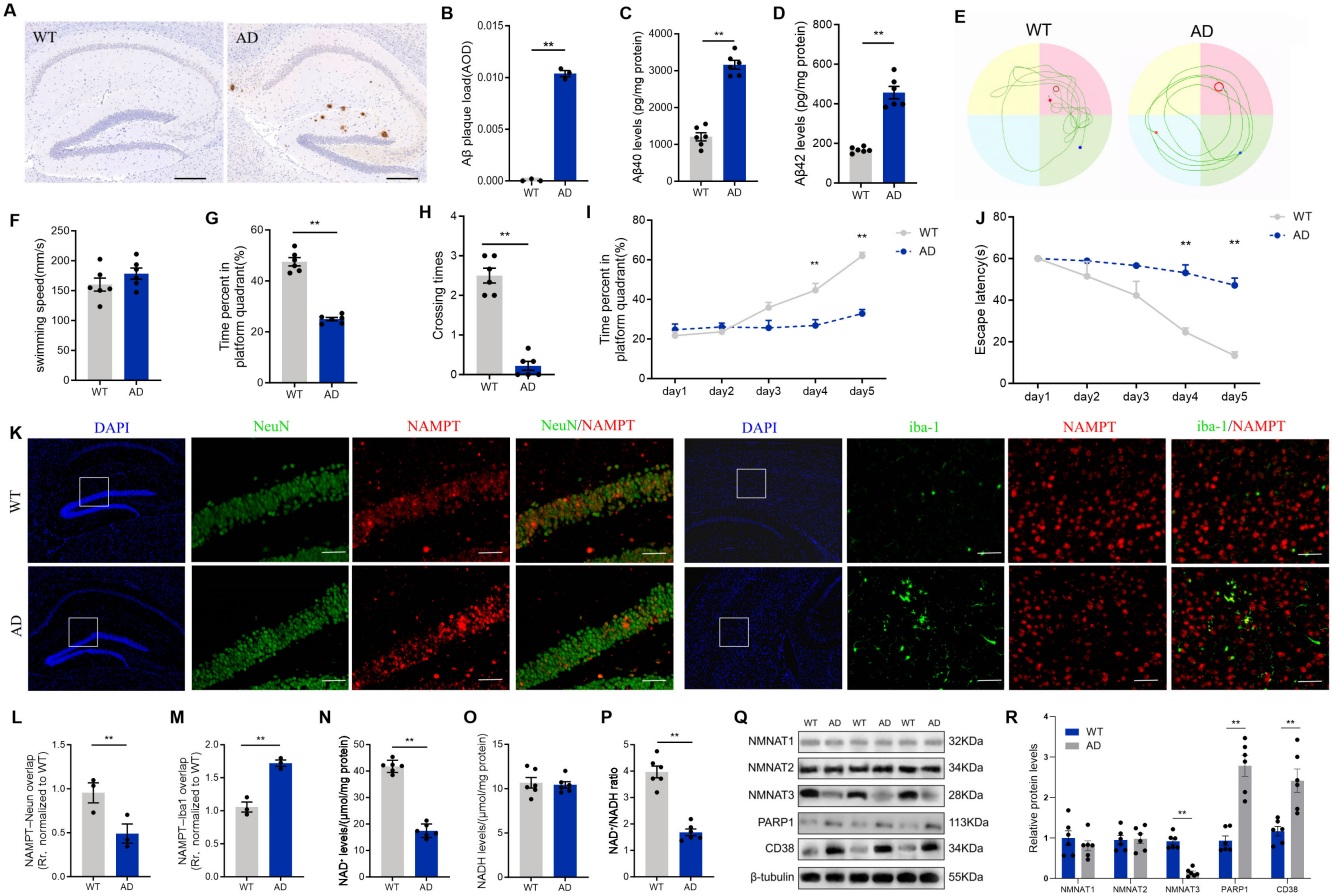
Cognitive performance and Aβ accumulation in APP/PS1 mice. **(A)** Immunohistochemical analysis showing Aβ plaques. Scale bar = 100 μm. **(B)** Quantitative analysis of Aβ plaque burden. **(C)** Aβ_40_ levels. **(D)** Aβ_42_ levels. **(E)** MWM spatial exploration trajectory map. **(F)** Average swimming speed in the visible platform trial. **(G)** Time spent in the platform quadrant during the probe trial. **(H)** Crossing times during the probe trial. **(I)** Time spent in the platform quadrant during the MWM learning phase. **(J)** Escape latency in the MWM learning phase. **(K)** Representative immunofluorescence images showing colocalization of NAMPT with NeuN and Iba-1 in the hippocampus. DAPI, blue; NeuN/Iba-1, green; NAMPT, red. **(L)** Quantitative analysis of NAMPT-NeuN overlap. **(M)** Quantitative analysis of NAMPT-Iba-1 overlap. **(N)** NAD^+^ levels. **(O)** NADH levels. **(P)** NAD^+^/NADH ratio. **(Q)** Representative Western blot bands of NMNAT1, NMNAT2, NMNAT3, PARP1, and CD38. **(R)** Quantitative analysis of relative protein levels. ** indicates a significant difference compared to the WT group (*P< 0.01*). Sample size: n=3 for **(A, B, K, L, M)**; n=6 for all others.

To characterize the basal pathological features of the APP/PS1 mice used in the present study, we first validated their AD-like phenotypes (**Figures 1A–J**). As shown in **Figures 1A–J**, compared with the WT group, 6-month-old APP/PS1 mice exhibited markedly increased Aβ deposition and a significantly higher Aβ plaque burden in the hippocampus. In the MWM test, APP/PS1 mice showed prolonged escape latency, fewer platform crossings, and reduced time spent in the target quadrant, with no significant difference in swimming speed observed. These results indicate that the APP/PS1 mice used in this study displayed stable AD-like pathological and behavioral abnormalities.

On this basis, to further characterise the abnormal expression pattern of NAMPT in the brains of APP/PS1 mice, we first examined the changes in NAMPT localization in different cell types. NeuN and Iba-1 were used to label neurons and microglia, respectively (23). Immunofluorescence colocalization analysis showed that, compared with the WT group, the colocalization of NAMPT with NeuN was markedly decreased, whereas the colocalization of NAMPT with Iba-1 was significantly increased in APP/PS1 mice (**Figures 1K–M**). These results suggest that NAMPT expression in the brains of APP/PS1 mice does not change uniformly but instead exhibits a distinct cell type-specific distribution pattern, characterized by reduced neuron-associated NAMPT signals and increased microglia-associated NAMPT signals.

Given that NAMPT is the key rate-limiting enzyme in the NAD^+^ salvage pathway (24), it remained to be determined whether this abnormal cellular distribution could affect cerebral NAD^+^ metabolic status. Therefore, we further measured the levels of NAD^+^, NADH, and the NAD^+^/NADH ratio in the hippocampus of APP/PS1 mice (**Figures 1N–P**). The results showed that, compared with the WT group, hippocampal NAD^+^ levels were significantly decreased in the AD group, whereas NADH levels were not significantly changed, and the NAD^+^/NADH ratio was significantly reduced, indicating that APP/PS1 mice exhibited marked NAD^+^ metabolic disturbance in the brain.

Since cerebral NAD^+^ homeostasis is regulated not only by NAMPT, but also by the coordinated actions of NAD^+^ biosynthetic and consuming enzymes (25), we further examined the expression of NAD^+^-related metabolic enzymes (**Figures 1Q, R**). Under physiological conditions, NAD^+^ levels are maintained by a dynamic balance between biosynthesis and consumption. The NMNAT family comprises key enzymes catalyzing the adenylyltransferase step in NAD^+^ biosynthesis, among which NMNAT1, NMNAT2, and NMNAT3 contribute to NAD^+^ generation (26). In addition, PARP1 and CD38 are important NAD^+^ consuming enzymes that are closely involved in NAD^+^ depletion (27). The results showed that, compared with the WT group, the protein expression levels of NMNAT1 and NMNAT2 in the hippocampus of APP/PS1 mice were not significantly altered, whereas NMNAT3 expression was significantly decreased. The protein expression levels of the NAD^+^ consuming enzymes PARP1 and CD38 were significantly increased. These results indicate that abnormal NAD^+^ metabolism exists in the hippocampus of APP/PS1 mice, which may be associated with NMNAT3 downregulation and enhanced NAD^+^ consumption mediated by PARP1 and CD38. Taken together, these findings suggest that the hippocampus of APP/PS1 mice exhibits NAD^+^ metabolic imbalance characterized by NAMPT abnormality, NMNAT3 downregulation, and increased PARP1 and CD38-associated consumption, implying that NAMPT-related NAD^+^ metabolic disturbance may participate in the subsequent pathological progression of AD.

### 3.2 APP/PS1 mice exhibit marked neuroinflammatory activation and mitochondrial structural and functional impairment in the hippocampus

**Figure 2:**
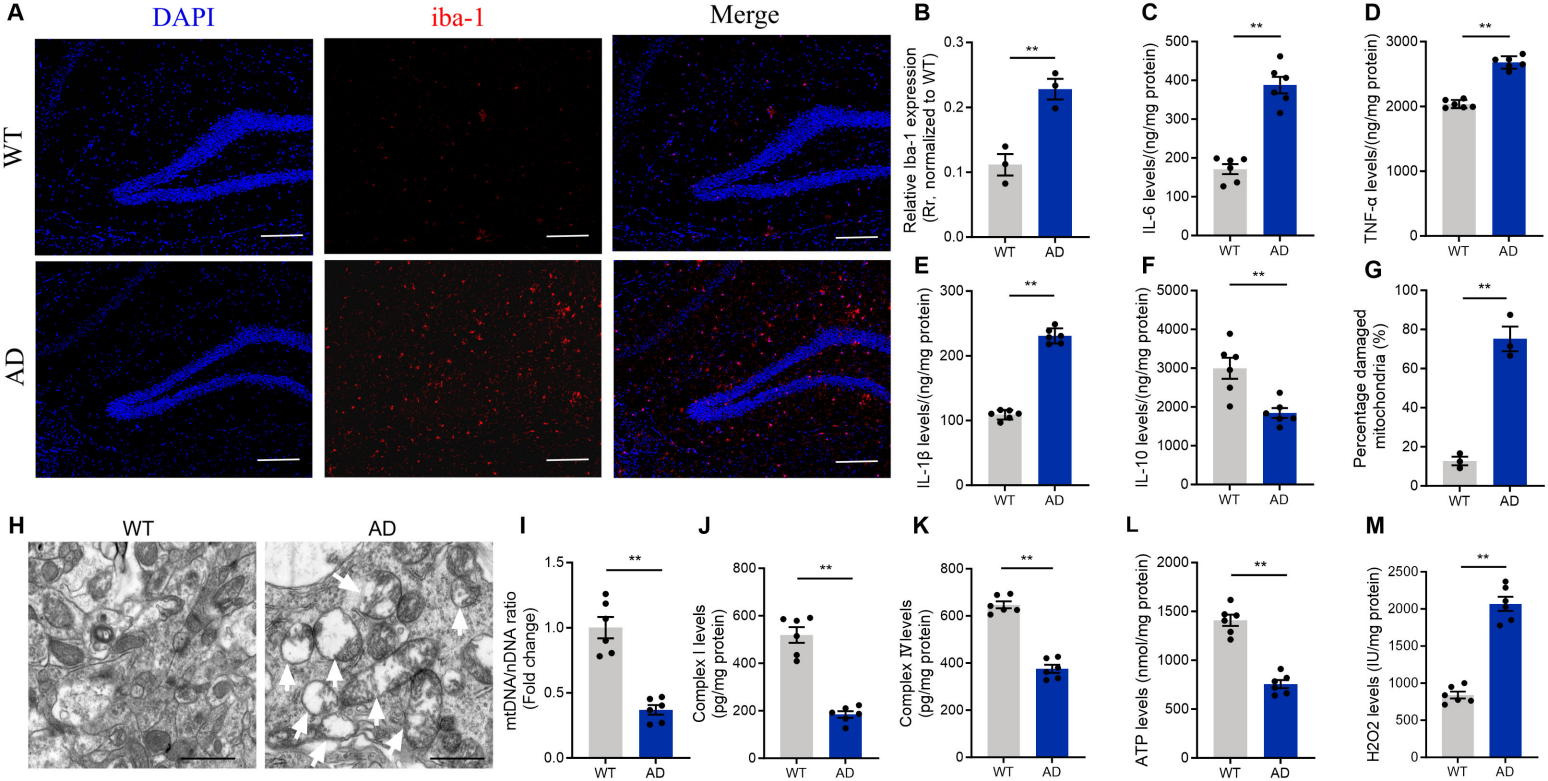
Neuroinflammation and mitochondrial dysfunction in APP/PS1 mice. **(A)** Immunofluorescence staining for Iba-1 **(red)** and DAPI (blue). **(B)** Relative Iba-1 expression. **(C)** IL-6 levels. **(D)** TNF-α levels. **(E)** IL-1β levels. **(F)** IL-10 levels. **(G)** Percentage of damaged mitochondria. **(H)** Transmission electron microscopy images of mitochondria. White arrows indicate mitochondria with obvious morphological damage. Scale bar = 2 μm. **(I)** mtDNA/nDNA ratio. **(J)** Levels of mitochondrial complex **(I, K)** Levels of mitochondrial complex IV. **(L)** ATP levels. **(M)** H_2_O_2_ levels. ** indicates a significant difference compared to the WT group (*P< 0.01*). Sample size: n = 3 for **(A, B, H, I)**; n = 6 for all others.

To further determine the pathological consequences associated with NAMPT-related NAD^+^ metabolic imbalance, we assessed changes in neuroinflammation- and mitochondrial function-related indices in the hippocampus of APP/PS1 mice (**Figures 2A–F**). The results showed that, compared with the WT group, Iba-1 immunoreactivity was significantly increased in the hippocampus of APP/PS1 mice, and microglia displayed a more activated morphology. ELISA results showed that the levels of the proinflammatory cytokines IL-6, TNF-α, and IL-1β were significantly elevated, whereas the level of the anti-inflammatory cytokine IL-10 was significantly decreased. These results indicate the presence of marked neuroinflammatory activation in the hippocampus of APP/PS1 mice.

In addition, we further examined mitochondrial structural and functional changes in the hippocampus of APP/PS1 mice (**Figures 2G–M**). Compared with the WT group, mitochondria in the hippocampus of APP/PS1 mice exhibited swelling, disrupted or blurred cristae, and vacuole-like changes, accompanied by a significantly increased proportion of damaged mitochondria. At the same time, hippocampal ATP levels were significantly decreased, the activities of Complex I and Complex IV were markedly reduced, H_2_O_2_ levels were significantly increased, and the mtDNA/nDNA ratio was significantly decreased in the AD group.

These results suggest that APP/PS1 mice exhibit marked mitochondrial structural damage and dysfunction in energy metabolism in the hippocampus. Taken together, these findings indicate that, on the basis of NAMPT-related NAD^+^ metabolic disturbance, the hippocampus of APP/PS1 mice exhibits pronounced neuroinflammatory activation and mitochondrial dysfunction, suggesting that both may contribute to the pathological progression of AD.

### 3.3 Exercise combined with FK866 ameliorates cognitive impairment and attenuates Aβ pathology in APP/PS1 mice

**Figure 3:**
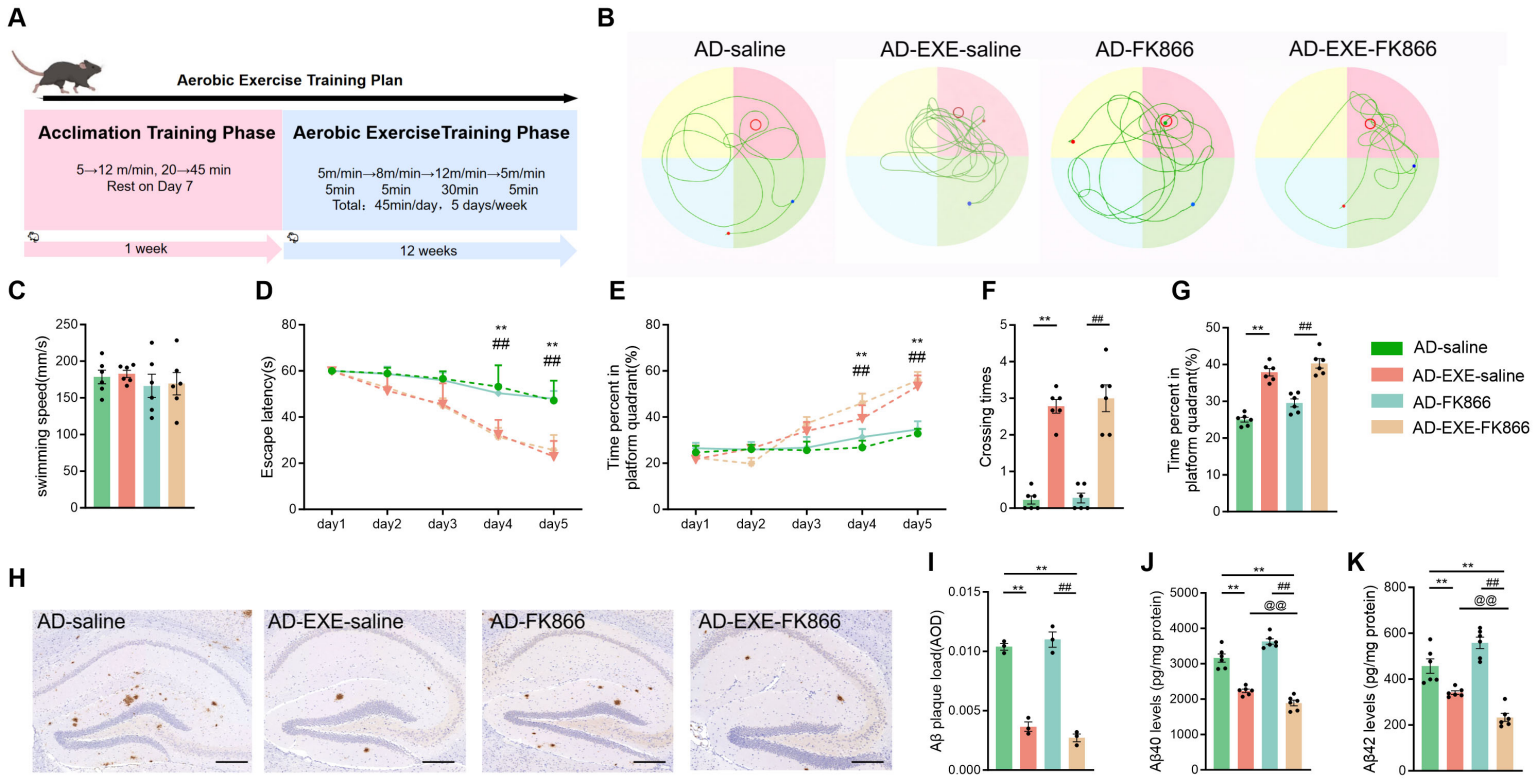
Effects of exercise and FK866 on cognitive function and Aβ accumulation. **(A)** Aerobic exercise training plan. **(B)** Representative swimming trajectories of mice during the Morris water maze probe trial (day 6). **(C)** Average swimming speed in the visible platform trial. **(D)** Escape latency in the MWM learning phase. **(E)** Time spent in the platform quadrant during the MWM acquisition phase. **(F)** Number of platform crossings during the probe trial. **(G)** Percentage of time spent in the platform quadrant during the probe trial. **(H)** Immunohistochemical staining showing Aβ plaque deposition. Scale bar = 100 μm. **(I)** Quantification of Aβ plaque burden. **(J)** Aβ_40_ levels. **(K)** Aβ_42_ levels. **Indicates a significant difference compared to the AD-saline group (*P< 0.01*). ## Indicates a significant difference compared to the AD-FK866 group (*P< 0.01*). @@ Indicates a significant difference compared to the AD-EXE-saline group (*P< 0.01*). Sample size: n = 3 for **(H, I)**, n = 6 for all others.

After confirming that APP/PS1 mice exhibited typical AD-like pathology, NAMPT-related NAD^+^ metabolic imbalance, neuroinflammatory activation, and mitochondrial dysfunction, we next evaluated the effects of exercise and FK866 on the overall pathological phenotype of APP/PS1 mice. The treadmill exercise protocol is illustrated in **Figure 3A**.

First, the Morris water maze test was used to assess learning and memory ability in each group of mice (**Figures 3B–G**). The results showed that there was no significant difference in swimming speed among the groups, suggesting that the different interventions did not markedly affect basal motor ability. Compared with the AD-saline group, APP/PS1 mice in the AD-EXE-saline group showed significantly shorter escape latency in the place navigation test, together with a marked increase in the number of platform crossings and time spent in the target quadrant during the probe test. Similar improvements were also observed in the AD-EXE-FK866 group, whereas cognitive function was not significantly improved in the AD-FK866 group. These results suggest that exercise ameliorates learning and memory impairment in APP/PS1 mice, whereas FK866 alone exerts only limited effects.

Given that Aβ pathological burden is an important pathological basis for learning and memory impairment (28), we further measured hippocampal Aβ plaque burden as well as Aβ_40_ and Aβ_42_ levels in each group (**Figures 3H–K**). Compared with the AD-saline group, the AD-EXE-saline group showed a marked reduction in hippocampal Aβ plaque burden, accompanied by significantly decreased levels of Aβ_40_ and Aβ_42_, whereas no significant improvement was observed in the AD-FK866 group. Notably, hippocampal Aβ_40_ and Aβ_42_ levels in the AD-EXE-FK866 group were significantly lower than those in the AD-EXE-saline group and remained at relatively low levels. These results indicate that exercise attenuates the Aβ pathological burden in the hippocampus of APP/PS1 mice. Compared with exercise alone, the combined intervention further reduced soluble Aβ_40_ and Aβ_42_ levels.

### 3.4 Exercise combined with FK866 suppresses neuroinflammatory responses and improves mitochondrial dysfunction in the hippocampus of APP/PS1 mice

**Figure 4:**
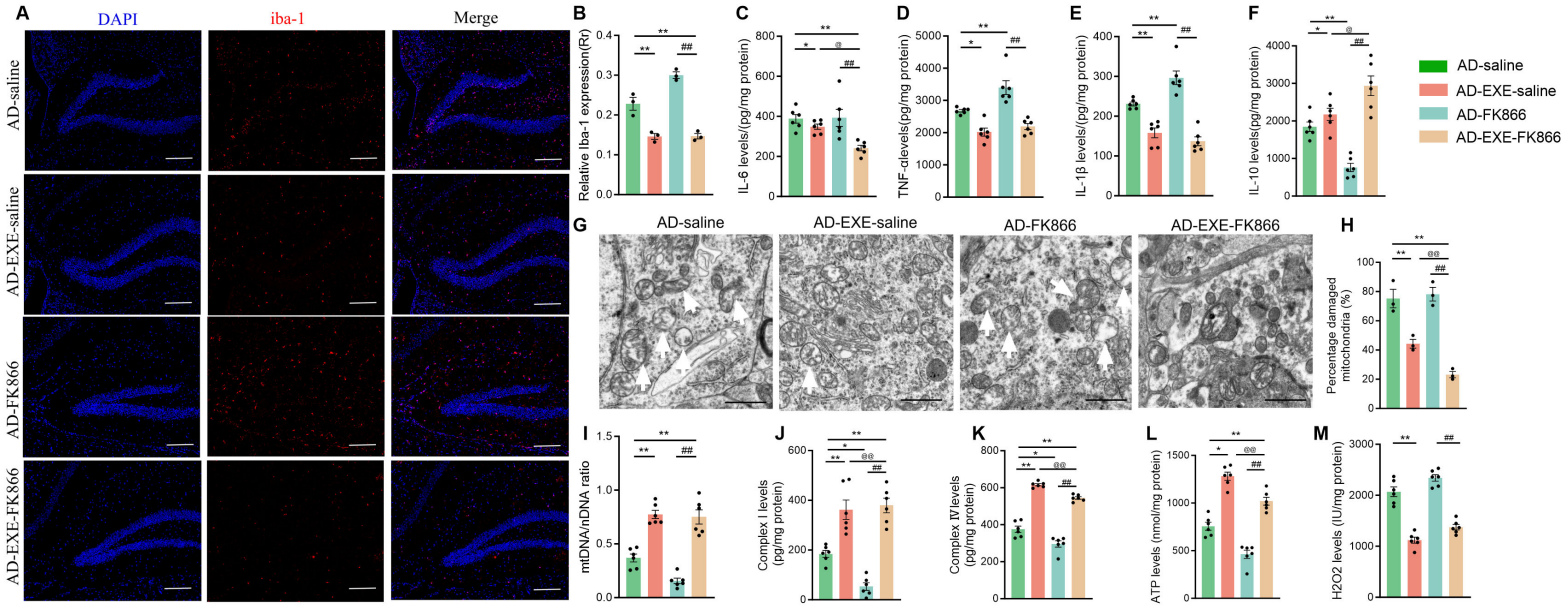
Effects of exercise and FK866 on neuroinflammation and mitochondrial function. **(A)** Immunofluorescence staining for Iba-1 (red) and DAPI (blue). Scale bar = 20 μm. **(B)** Relative Iba-1 expression. **(C)** IL-6 levels. **(D)** TNF-α levels. **(E)** IL-1β levels. **(F)** IL-10 levels. **(G)** Transmission electron microscopy images of mitochondria. White arrows indicate mitochondria with obvious morphological damage. Scale bar = 2 μm. **(H)** Percentage of damaged mitochondria. **(I)** mtDNA/nDNA ratio. **(J)** Levels of mitochondrial complex **(I, K)** Levels of mitochondrial complex IV. **(L)** ATP levels. **(M)** H_2_O_2_ levels. *Indicates a significant difference compared to the AD-saline group (*P< 0.05*). ** Indicates a significant difference compared to the AD-saline group (*P< 0.01*). ## Indicates a significant difference compared to the AD-FK866 group (*P< 0.01*). @ Indicates a significant difference compared to the AD-EXE-saline group (*P< 0.05*). @@ Indicates a significant difference compared to the AD-EXE-saline group (*P< 0.01*). Sample size: n = 3 for **(A, B, G, H)**; n = 6 for all other panels.

To further determine whether NAMPT-related neuroinflammation contributes to the beneficial effects of exercise combined with FK866 on AD-like pathological changes in APP/PS1 mice, we assessed microglial activation and the expression of inflammatory cytokines in the hippocampus of each group (**Figures 4A–F**). The results showed that, compared with the AD-saline group, Iba-1 immunoreactivity was markedly reduced in the hippocampus of the AD-EXE-saline group, suggesting that exercise suppresses microglial activation. A similarly reduced level of Iba-1 immunoreactivity was also observed in the AD-EXE-FK866 group. ELISA results showed that, compared with the AD-saline group, the levels of IL-6, TNF-α, and IL-1β were significantly decreased, whereas the level of the anti-inflammatory cytokine IL-10 was significantly increased in the hippocampus of the AD-EXE-saline group. Notably, compared with the AD-EXE-saline group, hippocampal IL-6 levels were further decreased, whereas IL-10 levels were further increased in the AD-EXE-FK866 group. These results indicate that exercise alleviates neuroinflammatory responses in the hippocampus of APP/PS1 mice and that FK866 combined with exercise exerts additional beneficial effects on neuroinflammation-related indices.

In addition to NAMPT-related neuroinflammatory activation, the influence of NAMPT-associated NAD^+^ metabolic imbalance on mitochondrial homeostasis may also be involved in the beneficial effects of exercise combined with FK866 on AD-like pathology in APP/PS1 mice. Therefore, we examined changes in mitochondrial ultrastructure and function in the hippocampus of each group (**Figures 4G–M**). Compared with the AD-saline group, mitochondrial damage in the hippocampus was significantly attenuated in the AD-EXE-saline group. Notably, mitochondrial ultrastructure was also markedly improved in the AD-EXE-FK866 group, and the proportion of damaged mitochondria was significantly lower than that in the AD-EXE-saline group. Further analyses showed that, compared with the AD-saline group, the *mtDNA/nDNA* ratio, activities of Complex I, Complex IV, and ATP levels were significantly increased, whereas H_2_O_2_ levels were significantly decreased in the AD-EXE-saline group. Notably, compared with the AD-EXE-saline group, H_2_O_2_ levels were further reduced in the AD-EXE-FK866 group. These results indicate that exercise markedly improves mitochondrial structural and functional impairment in the hippocampus of APP/PS1 mice and that, compared with exercise alone, the combined intervention further reduces the proportion of damaged mitochondria and H_2_O_2_ levels.

### 3.5 Exercise combined with FK866 remodels the hippocampal NAD^+^ metabolic environment in APP/PS1 mice

**Figure 5:**
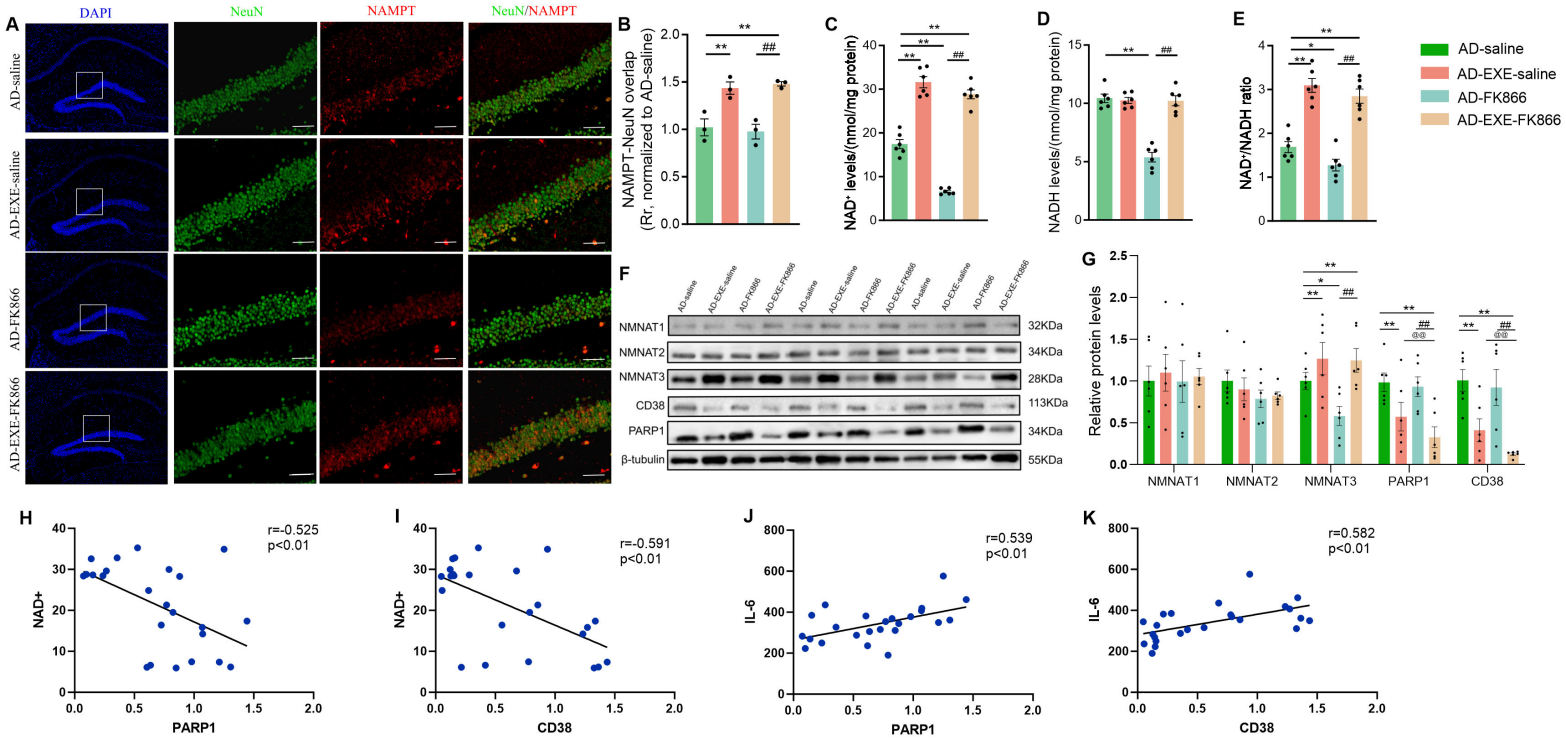
Effects of exercise and FK866 on hippocampal NAD^+^ metabolism. **(A)** Immunofluorescence staining for NeuN (green), NAMPT (red), and DAPI (blue). **(B)** Relative co-localization level of NeuN and NAMPT. **(C)** NAD^+^ levels. **(D)** NADH levels. **(E)** NAD^+^/NADH ratio. **(F)** Representative Western blotting images of NMNAT1, NMNAT2, NMNAT3, CD38, and PARP1 in the hippocampus, with β-tubulin used as the loading control. **(G)** Quantitative analysis of NMNAT1, NMNAT2, NMNAT3, CD38, and PARP1 protein expression. **(H)** Correlation analysis between PARP1 and NAD^+^ levels. **(I)** Correlation analysis between CD38 and NAD^+^ levels. **(J)** Correlation analysis between PARP1 and IL-6 levels. **(K)** Correlation analysis between CD38 and IL-6 levels. *Indicates a significant difference compared to the AD-saline group (*P< 0.05*). ** Indicates a significant difference compared to the AD-saline group (*P< 0.01*). ## Indicates a significant difference compared to the AD-FK866 group (*P< 0.01*). @@ Indicates a significant difference compared to the AD-EXE-saline group (*P< 0.01*). Sample size: n = 3 for **(A, B)**; n = 6 for **(C–G)**. n = 24 for **(H–K)**.

To further explore the potential mechanisms underlying the protective effects of exercise combined with FK866, we first examined the distribution of NAMPT in different cell types and the changes in NAD^+^ levels in the hippocampus of each group (**Figures 5A–E**). The results showed that, compared with the AD-saline group, the colocalization of NAMPT with NeuN was markedly increased in both the AD-EXE-saline and AD-EXE-FK866 groups. Notably, no significant difference was observed between the AD-EXE-saline and AD-EXE-FK866 groups, suggesting that exercise may be the major factor responsible for restoring neuronal NAMPT expression. ELISA results further showed that, compared with the AD-saline group, both hippocampal NAD^+^ levels and the NAD^+^/NADH ratio were significantly increased in the AD-EXE-saline and AD-EXE-FK866 groups. These results indicate that exercise improves NAD^+^ metabolic homeostasis in the AD brain.

We next examined the expression of NAD^+^-related metabolic proteins (**Figures 5F, G**). The results showed that there were no significant differences in NMNAT1 or NMNAT2 protein expression among the groups, whereas NMNAT3 expression differed significantly. Compared with the AD-saline group, the AD-EXE-saline group demonstrated significantly increase in NMNAT3 expression; moreover, compared with that in the AD-EXE-saline group, NMNAT3 expression was further restored in the AD-EXE-FK866 group. Compared with that in the AD-saline group, hippocampal PARP1 and CD38 protein expression levels were significantly decreased in the AD-EXE-saline group, whereas no significant improvement was observed in the AD-FK866 group. Notably, PARP1 and CD38 levels in the AD-EXE-FK866 group were further reduced compared with those in the AD-EXE-saline group.

To further determine whether PARP1 and CD38 were associated not only with NAD^+^ depletion but also with neuroinflammatory activation, we performed correlation analyses using individual data from the APP/PS1 groups (**Figures 5H–K**). The results showed that PARP1 and CD38 were negatively correlated with hippocampal NAD^+^ levels, whereas both were positively correlated with IL-6 levels. These findings suggest that PARP1 and CD38 are closely associated with both NAD^+^ metabolic disturbance and neuroinflammatory activation in APP/PS1 mice.

Taken together, these results suggest that exercise improves the NAD^+^ metabolic environment in APP/PS1 mice, and that the combined intervention with FK866 may further enhance this effect by increasing NMNAT3 expression while suppressing PARP1 and CD38, thereby supporting NAD^+^ biosynthesis, reducing NAD^+^ consumption, and attenuating neuroinflammation-related activation.

## 4 Discussion

The present study demonstrated that exercise combined with FK866 ameliorated AD-related pathological changes in APP/PS1 mice and was accompanied by changes in NAD^+^-related metabolic indices. We found that APP/PS1 mice not only exhibited a typical increase in Aβ pathological burden and impaired learning and memory ability, but were also accompanied by abnormal cell type-specific distribution of NAMPT and NAD^+^ metabolic imbalance, together with marked neuroinflammatory activation and mitochondrial structural and functional impairment. On this basis, 12 weeks of aerobic exercise significantly improved cognitive function, attenuated Aβ pathological burden, and alleviated neuroinflammation and mitochondrial damage in APP/PS1 mice. In contrast, FK866 alone exerted only limited effects. More importantly, the combined intervention with exercise and FK866 still showed beneficial effects and further improved selected indices of amyloid pathology, inflammation, and oxidative stress. These findings suggest that the combined intervention may have therapeutic potential in ameliorating selected aspects of AD pathology, and this pattern may be related to changes in NAD^+^-related metabolic indices.

NAMPT, the rate-limiting enzyme in the NAD^+^ salvage pathway, appears to play a complex role in AD pathology. In the present study, NeuN and Iba-1 were used as representative markers to assess neuron- and microglia-associated changes in NAMPT distribution (29, 30). We found that the colocalization of NAMPT with NeuN was decreased, whereas its colocalization with Iba-1 was increased, suggesting that, under AD-like pathological conditions, NAMPT shifts from a distribution pattern more favorable for neuronal metabolic maintenance to an abnormal distribution more closely associated with inflammation-related cells. Given the established role of NAMPT in maintaining NAD^+^ metabolism, the reduced neuron-associated NAMPT signals observed in APP/PS1 mice may be related to the concomitant NAD^+^ metabolic imbalance and mitochondrial impairment in the hippocampus. This possibility is supported by previous evidence showing that NAMPT depletion disrupts mitochondrial homeostasis and promotes neuronal degeneration in the mouse hippocampus (6). In addition, we observed increased protein expression of the NAD^+^ consuming enzymes PARP1 and CD38 in the hippocampus of 6-month-old APP/PS1 mice. Consistent with previous studies (10, 11), these findings suggest that imbalance in NAD^+^ metabolic homeostasis in the hippocampus of APP/PS1 mice is related not only to alterations in NAD^+^ biosynthetic enzymes, but also to increased protein expression of NAD^+^ consuming enzymes. Notably, this metabolic disturbance was accompanied by marked neuroinflammatory activation and mitochondrial structural and functional impairment, as reflected by inflammatory cytokine imbalance, disrupted mitochondrial ultrastructure, and reduced indices of energy metabolism. Together, these findings suggest that NAMPT-related NAD^+^ metabolic disturbance may be closely associated with neuroinflammatory activation, mitochondrial dysfunction, and pathological progression in the AD brain.

Against this pathological background, the present study further demonstrated that 12 weeks of aerobic exercise not only significantly improved learning and memory ability in APP/PS1 mice, but also reduced hippocampal Aβ plaque burden and decreased Aβ_40_ and Aβ_42_ levels. This is consistent with previous studies showing that regular exercise can delay AD-like pathological progression, ameliorate cognitive impairment, and reduce Aβ deposition (31). In addition, we found that exercise suppressed microglial activation and improves the inflammatory imbalance reflected by IL-6, TNF-α, IL-1β, and IL-10, which is also in agreement with previous studies suggesting that exercise can modulate the cerebral inflammatory environment and attenuate neuroinflammatory responses (32). Furthermore, exercise intervention markedly improved mitochondrial ultrastructure, increased ATP levels and the activities of Complex I and Complex IV, and decreased H2O2 levels in the hippocampus of APP/PS1 mice, indicating that exercise exerts a clear protective effect on mitochondrial structural integrity and energy metabolism. This finding is consistent with those of previous reports showing that exercise improves mitochondrial biogenesis, oxidative phosphorylation, and oxidative stress status (33). Notably, the present study also showed that exercise increased hippocampal NAD^+^ levels and the NAD^+^/NADH ratio, downregulated PARP1 and CD38 expression, and ameliorated the abnormal distribution of NAMPT among different cell types. Together with previous studies showing that exercise can activate NAD^+^-dependent metabolic pathways, enhance cellular metabolic adaptability, and maintain cerebral homeostasis (34), our findings suggest that exercise directly improves the pathological phenotype of APP/PS1 mice and is accompanied by improvement in cerebral metabolic homeostasis.

Most importantly, the present study further showed that the combined intervention with exercise and FK866 did not diminish the protective effects of exercise, but instead conferred additional improvements in selected pathological indices. Compared with the exercise-alone group, the combined intervention group showed further reductions in Aβ_40_ and Aβ_42_ levels, lower IL-6 levels, higher IL-10 levels, and a significant decrease in H_2_O_2_ levels. These findings indicate that FK866, when combined with exercise, does not weaken the beneficial effects of exercise, but instead may further improve selected pathological outcomes. Although direct evidence is still lacking, we speculate that this may be related to the dual role of NAMPT in the AD inflammatory microenvironment, where NAMPT may not only participate in NAD^+^ biosynthesis but also be involved in inflammation-related glial responses. Therefore, FK866 may partially suppress inflammation-associated NAMPT signaling (4, 35), whereas exercise may help maintain metabolic adaptability and mitochondrial homeostasis (36). This complementary pattern may help explain why the combined intervention produced further improvements in inflammatory and oxidative stress-related indices.

In addition, the present study showed that exercise increased hippocampal NAD^+^ levels and the NAD^+^/NADH ratio in APP/PS1 mice, accompanied by increased NMNAT3 expression and decreased PARP1 and CD38 expression, which is consistent with previous studies showing that exercise improves NAD^+^ metabolic status and metabolic homeostasis (34, 37). Compared with the exercise-alone group, the combined intervention group showed further increase in NMNAT3 expression, together with further reduction in PARP1 and CD38 expression. Previous studies have shown that PARP1 and CD38 are closely associated with NAD^+^ depletion, neuroinflammation, and impaired energy metabolism (38). Therefore, the superior effect of the combined intervention over exercise alone may be associated with enhanced NAD^+^ biosynthesis, as reflected by increased NMNAT3 expression, as well as reduced PARP1 and CD38-related NAD^+^ consumption and neuroinflammation-related activation.

Similar additive benefits of combined interventions have also been reported in other AD models. Previous studies have shown that, in 3×Tg-AD mice, exercise combined with melatonin is more effective than exercise alone in maintaining mitochondrial complex levels (39). Likewise, in APP/PS1 mice, exercise combined with chlorogenic acid has been reported to exert more pronounced beneficial effects on oxidative stress, neuroinflammation, and AD pathology than exercise alone (40). Consistent with these observations, the present findings support the notion that combining exercise with interventions that exert partially complementary effects may confer additional benefits over exercise alone in improving selected pathological outcomes.

Taken together, the present study showed that APP/PS1 mice exhibit an abnormal cell type-specific distribution of NAMPT and disrupted NAD^+^ metabolic homeostasis in the brain, accompanied by marked neuroinflammatory activation and mitochondrial dysfunction, further supporting a critical role of NAD^+^ metabolic disturbance in the pathological progression of AD. On this basis, we found that aerobic exercise significantly ameliorated AD-like pathological changes in APP/PS1 mice, whereas FK866 alone exerted limited effects. Importantly, the combined intervention of exercise and FK866 conferred clear protective effects and, compared with exercise alone, provided additional improvements in selected indices related to amyloid pathology, inflammation, and oxidative stress. These findings suggest that the superior effect of the combined intervention may be associated with enhanced NAD^+^ biosynthesis, as indicated by increased NMNAT3, together with reduced PARP1/CD38-associated NAD^+^ consumption and neuroinflammation-related activation. Compared with FK866 alone, the combined intervention may offer greater therapeutic potential in improving selected aspects of AD-like pathology.

## 5 Conclusion

The present study identified abnormal cell type-specific distribution of NAMPT as a feature of disrupted hippocampal NAD^+^ metabolic homeostasis in APP/PS1 mice, accompanied by marked neuroinflammatory activation, mitochondrial dysfunction, amyloid pathology, and cognitive impairment. Against this pathological background, aerobic exercise significantly ameliorated AD-like pathological changes in APP/PS1 mice, whereas FK866 alone exerted only limited effects. Importantly, the combined intervention with exercise and FK866 also showed clear protective effects and, compared with exercise alone, produced additional improvements in selected indices related to amyloid pathology, inflammation, and oxidative stress. These findings suggest that the superior effect of the combined intervention may be associated with increased NAD^+^ biosynthesis through the NMNAT3 pathway, together with reduced NAD^+^ consumption and neuroinflammatory activation through suppression of CD38 and PARP1 expression. This study suggests that the beneficial effects of FK866 may depend on a relatively stable NAD^+^ metabolic background. When combined with interventions that maintain NAD^+^ homeostasis, FK866 shows greater potential to ameliorate AD-related pathology, thereby providing experimental support for optimizing NAMPT-targeted strategies in AD through the maintenance of metabolic homeostasis.

### 5.1 Research limitations

NAMPT distribution was examined in hippocampal NeuN-positive neurons and Iba-1-positive microglia. Whether similar changes occur in astrocytes or other brain cell populations remains to be determined. In addition, cognitive performance was assessed using the Morris water maze alone, and the possible influence of anxiety-related behavior cannot be fully excluded. Future studies incorporating cell-type-specific analyses together with non-aversive memory tests will be needed to address these issues.

## Data Availability

The original contributions presented in the study are included in the article/supplementary material. Further inquiries can be directed to the corresponding author.
